# Solving Coupled Cluster Equations by the Newton Krylov Method

**DOI:** 10.3389/fchem.2020.590184

**Published:** 2020-12-10

**Authors:** Chao Yang, Jiri Brabec, Libor Veis, David B. Williams-Young, Karol Kowalski

**Affiliations:** ^1^Computational Research Division, Lawrence Berkeley National Laboratory, Berkeley, CA, United States; ^2^J. Heyrovsky Institute of Physical Chemistry, Academy of Sciences, Prague, Czechia; ^3^Pacific Northwest National Laboratory, Richland, WA, United States

**Keywords:** couple cluster approximation, Newton-Krylov method, DIIS, precondition, nonlinear solver

## Abstract

We describe using the Newton Krylov method to solve the coupled cluster equation. The method uses a Krylov iterative method to compute the Newton correction to the approximate coupled cluster amplitude. The multiplication of the Jacobian with a vector, which is required in each step of a Krylov iterative method such as the Generalized Minimum Residual (GMRES) method, is carried out through a finite difference approximation, and requires an additional residual evaluation. The overall cost of the method is determined by the sum of the inner Krylov and outer Newton iterations. We discuss the termination criterion used for the inner iteration and show how to apply pre-conditioners to accelerate convergence. We will also examine the use of regularization technique to improve the stability of convergence and compare the method with the widely used direct inversion of iterative subspace (DIIS) methods through numerical examples.

## 1. Introduction

The coupled cluster (CC) theory, introduced to quantum chemistry by Čížek (1966), Paldus and Li (1999), and Bartlett and Musiał (2007), over the past few decades has established itself as one of the most accurate *ab initio* method for electronic structure calculations. The systematic inclusion of higher-rank excitations in the cluster operator allows one to establish a hierarchy of more and more accurate approximations converging toward the full configuration interaction (FCI) limit (Gauss, 1998). These standard approximations also provide a number of unique features such as size-extensivity of the resulting energies, orbital invariance of theory under separate rotations of occupied and virtual orbitals, the possibility of approximating higher excitations by products of lower-rank clusters, which are especially important in proper description of chemical transformations associated with bond forming and bond breaking processes. The CC theory is based on an exponential ansatz acting on the reference wave function, typically Slater determinant obtained from Hartree–Fock (HF), density functional theory, or other independent particle models, which is assumed to provide a reasonable zeroth order description of the correlated ground-state wave function. The CC wave function is determined by the so-called cluster amplitudes obtained by solving nonlinear energy-independent CC equations.

The exponential ansatz ensures the size-extensivity of the CC method, but in contrast to configuration interaction methods, the CC method is not variational (unless all excitations are included). In practice, to make CC approximation numerically feasible, the cluster operator is defined by low-rank excitations. For example, one of the most widely used coupled cluster single and double (CCSD) model includes single and double excitations (Purvis and Bartlett, 1982). The single-reference CCSD method and non-iterative technique for the inclusion of collective triple excitations [the so-called CCSD(T) approach; Raghavachari et al., 1989] is currently considered as a “gold standard” of high-accuracy computational chemistry. It is available in many program packages and employed widely in a wide variety of chemical applications. The numerical cost scales polynomially with the system size, where the numerical scaling of CCSD is proportional to $O\left(N^{6}\right)$, whereas for (T) correction is proportional to $O\left(N^{7}\right)$ (*N* represents symbolically system size).

For the treatment of the static correlation effects, the multireference CC approach has been introduced, which generalizes the CC exponential parameterization of the wave function (Lyakh et al., 2012). Out of many formulations of MRCC theories, the class of methods relevant to this work is externally corrected CC, which extracts information about the most important higher excitations or active space single and double excitations from an “external” calculation performed by a different method such as complete active space self-consistent field (CASSCF) or multireference configuration interaction (MRCI) (Li and Paldus, 1997; Li, 2001; Kinoshita et al., 2005). In this work, we employed the tailored CCSD (TCCSD) method, where the information for external correction is obtained from a density matrix renormalization group (DMRG) calculation. The TCC approach has been successfully applied (Kinoshita et al., 2005; Lyakh et al., 2011) and generally performs well, although a large active space and CASSCF orbitals might be required for good accuracy. TCC also features the desirable property of being rigorously size extensive.

The CCSD equations correspond to the polynomial set of equations of fourth order, whose solving for large number of cluster amplitudes (often exceeding 10^10^) in the presence of strong correlation effects may pose a significant challenge and may adversely affect the time to solution associated with solving CC equations, even when efficient implementation of the CCSD method is available. Therefore, the design of fast converging CC solvers is inextricably linked to the effort of enabling CC methods at exa-scale. Currently, CC equations are typically solved via an inexact Newton (IN) method combined with an acceleration scheme called the direct inversion of iterative space (DIIS) (Pulay, 1980), which is also used in many other quantum chemical algorithms to accelerate the convergence, for example in the self-consistent field (SCF) iterations for solving the HF equations. Several other algorithms such as reduced linear equation (Purvis and Bartlett, 1981), quasilinearization of nonlinear terms techniques (Piecuch and Adamowicz, 1994), and multimodel Newton-type algorithms (Kjønstad et al., 2020) have been tested especially in the context of solving CC equations involving high-rank clusters.

In this paper, we describe using the Newton–Krylov (NK) method for solving the projected CC equation. The NK method is a widely used method for solving large-scale nonlinear equations in many fields (Knoll and Keyes, 2004). Its use in quantum chemistry appears to be new. We will describe the basic steps of the method in the context of CCSD in section 3. We compare the method with DIIS in section 4 and discuss the possibility of combining the two methods together. In section 5, we demonstrate the performance of the NK method and compare it with DIIS.

## 2. Coupled Cluster Equations

In this section, we briefly discuss the algebraic form of the CC equations for cluster amplitudes. In general, a correlated wave function |Ψ〉 can be written as,

(1) $$\begin{array}{l} |\text{Ψ}\rangle =\Omega |\Phi \rangle , \end{array}$$

where |Φ〉 is the reference wave function (typically the HF Slater determinant) and Ω is the wave operator. In the CC method, the wave operator is assumed in an exponential form

(2) $$\begin{array}{l} \Omega =e^{T}, \end{array}$$

where *T* is the cluster operator defined by excitations producing excited Slater determinant when acting on the reference function. This property of the cluster operator *T* assures the so-called intermediate normalization of the CC wave function, i.e.,

(3) $$\begin{array}{l} \langle \text{Ψ}|\Phi \rangle =1, \end{array}$$

assuming that orthonormal molecular basis set was used to discretize many-body problem of interest. The cluster operator *T* is a sum of its many-body components

(4) $$\begin{array}{l} T=T_{1}+T_{2}+\ldots , \end{array}$$

where *T*_*n*_ is the linear combination of excitation operators, which corresponds to *n*-tuple excitations. Thus, for single- and double-excitations one can write

(5) $$\begin{array}{lll} T_{1} & = & \underset{i,j}{\sum }t_{i}^{a}a_{a}^{†}a_{i} \end{array}$$

(6) $$\begin{array}{lll} T_{2} & = & \underset{i<j;a<b}{\sum }t_{ij}^{ab}a_{a}^{†}a_{b}^{†}a_{j}a_{i}, \end{array}$$

(7) $$\begin{array}{l} \ldots \end{array}$$

The coefficients $t_{ij..}^{ab..}$ are the cluster amplitudes, which will be determined by solving CC equations. We use the standard notation, i.e., indices *i, j* denote occupied, *a, b* virtual, and *p, q* general molecular spin orbitals. $a_{p}^{†}$ and *a*_*q*_ are fermionic creation or annihilation operators which satisfy set of anticommutation relations

(8) $$\begin{array}{l} \left\{a_{p}^{†},a_{q}\right\}=a_{p}^{†}a_{q}+a_{q}a_{p}^{†}=\delta_{pq} \end{array}$$

(9) $$\begin{array}{l} \left\{a_{p}^{†},a_{q}^{†}\right\}=\left\{a_{p},a_{q}\right\}=0. \end{array}$$

In the context of deriving algebraic form of CC equations, particle-hole formalism is invoked (Shavitt and Bartlett, 2009).

Inserting the CC ansatz into the Schrödinger equation and pre-multiplying from the left by *e*^−*T*^ yields

(10) $$\begin{array}{l} e^{-T}He^{T}\Phi =E\Phi . \end{array}$$

To employ various diagrammatic techniques to derive CC equations, it is useful to introduce the normal product form of the electronic Hamiltonian (*H*_*N*_) defined as

(11) $$\begin{array}{l} H_{N}=H-\langle \Phi |H|\Phi \rangle =\underset{pq}{\sum }F_{pq}N\left\{p^{†}q\right\}+\frac{1}{4}\underset{pqrs}{\sum }\langle pq\|rs\rangle N\left\{p^{†}q^{†}sr\right\}, \end{array}$$

Using the Baker–Campbell–Hausdorff formula, we get

(12) $$\begin{array}{l} e^{-T}H_{N}e^{T}=H_{N}+\left[H_{N},T\right]+\frac{1}{2}\left[\left[H_{N},T\right],T\right]+\\ \;+\frac{1}{3!}\left[\left[\left[H_{N},T\right],T\right],T\right]+\frac{1}{4!}\left[\left[\left[\left[H_{N},T\right],T\right],T\right],T\right]\\ \;\equiv {\left(H_{\text{N}}e^{T}\right)}_{\text{C}}, \end{array}$$

where subscript “C” corresponds to a connected part of a given operator expression. Since electronic Hamiltonians are defined by one- and two-body interactions, the above expansion terminates after quadruple commutator (11). For the derivation of the correlation energy expression and amplitude equations, we project the $e^{-T}H_{N}e^{T}|\Phi \rangle$ term to the bra vectors 〈Φ|, $\langle \Phi_{i}^{a}|$, $\langle \Phi_{ij}^{ab}|$, etc.:

(13) $$\begin{array}{l} \Delta E_{\text{corr}}={\langle \Phi |H_{N}e^{T}|\Phi \rangle }_{\text{C}}, \end{array}$$

(14) $$\begin{array}{l} {\langle \Phi_{ij\ldots }^{ab\ldots }|H_{N}e^{T}|\Phi \rangle }_{\text{C}}=0 \end{array}$$

where $\langle \Phi_{i}^{a}|,\langle \Phi_{ij}^{ab}|,\ldots$ represent singly, doubly, etc., excited Slater determinants with respect to the reference function.

The general TCC wave function employs the following split-amplitude ansatz

(15) $$\begin{array}{l} \Omega_{\text{TCC}}=e^{T^{\text{ext}}+T^{\text{act}}}, \end{array}$$

where *T*^act^ represents the active amplitudes obtained from the active space calculation. These amplitudes are kept constant when solving the amplitude equations, only *T*^ext^ are iterated. The *T*^act^ amplitudes are computed from the CI coefficients, extracted from the matrix product states wave function optimized during the DMRG calculation. We will use *t* to denote, collectively, the CCSD amplitudes in Equations (5) and (6) that are contained in *T*^ext^. These amplitudes satisfy a nonlinear equation that can be derived from Equation (14). In the rest of the paper, we will simply write this equation as,

(16) $$\begin{array}{l} r\left(t\right)=0. \end{array}$$

## 3. Algorithms for Solving the CCSD Equation

In this section, we begin with a short description of a general scheme for solving the CCSD nonlinear equation using an inexact Newton's method. We review a commonly used diagonal approximation to the Jacobian, and then describe the NK method for solving the CCSD equation.

### 3.1. Inexact Newton's Method

Even though Equation (16) is only a second-order nonlinear equation, it is not easy to solve due to the large number of variables contained in *t*. One should remember that in the state-of-the-art CCSD calculations, the total number of sought cluster amplitudes exceeds 10^10^. An iterative procedure is generally required to solve the equation numerically. The best known algorithm for solving a general system of nonlinear equations is the Newton's method. In the *k*+1st iteration of such a method, the approximation to the solution of Equation (16) is updated as

(17) $$\begin{array}{l} t^{\left(k+1\right)}=t^{\left(k\right)}-{\left[J^{\left(k\right)}\right]}^{-1}r\left(t^{\left(k\right)}\right), \end{array}$$

where *t*^(*k*)^ is the approximate solution obtained from the *k*th iteration, and *J*^(*k*)^ is the Jacobian of *r*(*t*) evaluated at *t*^(*k*)^.

Because it is not practical to write down the Jacobian of *r*(*t*) or its inverse analytically, we cannot use the Newton's method directly to solve the CC equation. Instead, an IN algorithm of the form

(18) $$\begin{array}{l} t^{\left(k+1\right)}=t^{\left(k\right)}-{\left[{Ĵ}^{\left(k\right)}\right]}^{-1}r\left(t^{\left(k\right)}\right) \end{array}$$

where Ĵ^(*k*)^ is an approximate Jacobian matrix evaluated at *t*^(*k*)^, is often used. In Equations (17) and (18), we view *t* and *r*(*t*) as a column vector with all amplitudes in (5) and (6) enumerated in some specific order, and the contracted tensor amplitudes in *r*(*t*) enumerated in the same order.

In CCSD calculation, a common practice is to choose Ĵ as a diagonal matrix with HF orbital energy difference as the diagonal elements. This is justified because *J* is known to be diagonal dominant in many cases, and the diagonal matrix of HF orbital energy differences contributes most to the diagonal of *J*. Replacing *J* with Ĵ typically works well when the system is near equilibrium. In this case, the computational cost of the IN method is dominated by the tensor contraction cost for evaluating *r*(*t*^(*k*)^) for each *k*, which has the complexity of *O*(*N*^6^) where *N* is the number of atomic basis used to discretize HF molecular orbitals.

For systems that do not satisfy this property, the diagonal approximation may not be sufficient. As a result, many IN iterations may be required to reach convergence, which is defined by the norm of *r*(*t*) being less than a prescribed tolerance level τ. This will lead to extremely long wall clock time.

### 3.2. Newton–Krylov Method

Even though *J* is not explicitly available, it is possible to approximate the product of *J*(*t*) with any tensor *w* that has the same dimension as *t*. This can be done through a finite difference calculation of the form

(19) $$\begin{array}{l} J\left(t\right)w\approx \frac{r\left(t+\delta w\right)-r\left(t\right)}{\delta }, \end{array}$$

where δ is a small constant.

The possibility to approximate *J*(*t*)*w* by one extra function evaluation allows us to solve the Newton correction equation

(20) $$\begin{array}{l} J\left(t^{\left(k\right)}\right)\Delta =-r\left(t^{\left(k\right)}\right), \end{array}$$

by a Krylov subspace-based iterative method such as the GMRES algorithm (Saad and Schultz, 1986) even when *J*(*t*) is not explicitly available. The solution Δ is used to update the approximate amplitude via

(21) $$\begin{array}{l} t^{\left(k+1\right)}=t^{\left(k\right)}+\Delta . \end{array}$$

This approach is often referred to as the NK method.

In Algorithm 1, we give a description of the simplest Newton-GMRES algorithm for solving the coupled clustered equation. We treat the CC amplitude *t* and tensors (*r*, *w*) of the same dimension as vectors, and denote the inner product of *t* and *r* simply as 〈*t, r*〉. We treat a set of tensors as a matrix, and use *V*(:, *j*), i.e., the *j*th column of *V*, to denote the *j*th tensor in such a set. The vector *e*_1_ used in this algorithm denotes a unit vector of length *j*_g_ + 1 with 1 in the first entry and 0 elsewhere.

**Algorithm 1:**
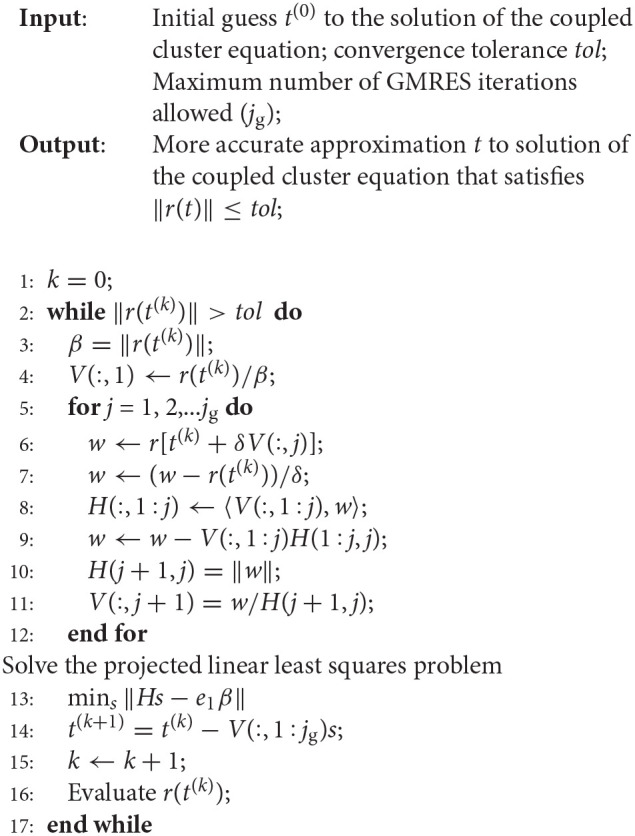
A Newton–Krylov method for solving the coupled cluster equation.

The outer *k* loop of Algorithm 1 performs the Newton update (21) in line 14, using Δ = *Vs* as the the approximate solution to the Newton correction equation, where *V* is an *n* × *j*_g_ matrix, where *n* is the total number of CCSD amplitudes, and *j*_g_ is the number of inner GMRES iterations. The inner *j* iteration of Algorithm 1 solves the Newton correction Equation (20) using the GMRES method. The GMRES method performs a Gram–Schmidt process to produce an orthonormal basis of the Krylvo subspace

$$K\left(J,r_{0}\right)\equiv \left\{r_{0},Jr_{0},J^{2}r_{0},\ldots ,J^{j_{\text{g}}}r_{0}\right\},$$

where *J* is the Jacobian evaluated at a particular approximation to the CCSD amplitudes *t*, and *r*_0_ is the function value of *r* in Equation (16) defined at such a *t*. This orthonormal basis is stored in columns of the *V* matrix. In exact arithmetic, this matrix satisfies the equation

(22) $$\begin{array}{l} JV=ṼH, \end{array}$$

where *V* contains the leading *j*_g_ columns of Ṽ, which are orthonormal, i.e., *V*^*T*^*V* = *I*, and *H* is a (*j*_g_ + 1) × *j*_g_ upper Hessenberg matrix. The approximation to the solution of (20) is represented as Δ = *Vs* for some vector *s* of length *j*_g_. This vector can be solved from the least squares problem defined by the Galerkin projection

(23) $$\begin{array}{l} \underset{s}{\min}\|{Ṽ}^{T}\left(JVs-r_{0}\right)\| \end{array}$$

It follows from Equation (22), Ṽ^*T*^Ṽ = *I*, and the fact that the first column of *V* is *r*_0_/||*r*_0_||, solving Equation (23) is equivalent to solving

(24) $$\begin{array}{l} \underset{s}{\min}\|Hs-\beta e_{1}\|, \end{array}$$

where β = ||*r*_0_||. This is the least squares problem solved on line 13 of the algorithm. The solution is used in line 14 to update the CCSD amplitude.

### 3.3. Precondition

An iterative procedure for computing the solution to the Newton correction Equation (20) can be accelerated by using a pre-conditioner *P*. Instead solving (20), we solve

(25) $$\begin{array}{l} P^{-1}J^{\left(k\right)}\Delta =-P^{-1}r\left(t^{\left(k\right)}\right), \end{array}$$

with the hope that *P*^−1^*J*^(*k*)^ has a reduced conditioner number. The reduced condition number can lead to faster convergence.

It is well-known that the Jacobian associated with the projected coupled cluster equation can be partitioned as

(26) $$\begin{array}{l} J\left(t^{\left(k\right)}\right)=D+E\left(t^{\left(k\right)}\right), \end{array}$$

where *D* is a diagonal matrix consisting of the difference between virtual and occupied HF orbital energies, and *E* is a complicated term that depends on the fluctuation potential (Helgaker et al., 2014). When the HF amplitude is relatively large, the first term is dominant. Hence the diagonal matrix can be used as a pre-conditioner for the iterative solver of Equation (20).

Applying such a pre-conditioner only requires adding an extra step before the **while** loop in Algorithm to compute the preconditioned right-hand side in Equation (25) and modifying line 7 of the algorithm to apply *P*^−1^ to the *W* tensor.

When *D* is ill conditioned due to the presence of near degenerate HF orbital energy levels, it may be necessary to introduce a shift σ and use *D*−σ*I* as the preconditioner. This *regularization* technique is similar to the level-shifting technique often used in HF SCF calculation (Saunders and Hillier, 1973).

When *J* is not dominated by *D*, i.e., when contribution from the HF amplitude is less significant, it is desirable to construct alternative pre-conditioners to accelerate the convergence of the NK method.

### 3.4. Stopping Criterion

It is well-known that fast convergence of the IN algorithm can be achieved even when the Newton correction Equation (20) is not solved to full accuracy (Eisenstat and Walker, 1994). This is especially true in earlier IN iterations in which the residual norm ||*r*(*t*)|| is still relatively large. A general strategy proposed in Eisenstat and Walker (1994) is to solve the correction equation to satisfy the following condition:

(27) $$\begin{array}{l} \|J\left(t^{\left(k\right)}\right)\Delta +r\left(t^{\left(k\right)}\right)\|\leq \eta_{k}\|r\left(t^{\left(k\right)}\right)\|, \end{array}$$

for some small constant 0 < η_*k*_ < 1. A sophisticated scheme was proposed in Eisenstat and Walker (1994) to ensure the so-called “global convergence.” However, such a scheme requires backtracking and additional residual function evaluations and is thus likely to increase the overall cost of the NK method. In this work, we use a simple strategy and set η_*k*_ to 10^−1^ with the left-hand side of Equation (27) estimated by the projected residual norm evaluated at the least squares solution to Equation (24). As the residual norm ||*r*(*t*^(*k*)^)|| decreases in the outer iteration, the absolute error in the approximate solution to the correction equation also decreases when Equation (27) is satisfied. Note that there is a trade-off between the number of inner GMRES iterations required to solve the Newton correction equation and the number of NK outer iterations. Setting η_*k*_ to a small number may result in too many GMRES iterations performed at each NK iteration and thus increase the overall cost the algorithm. In our implementation, we set a limit on the maximum number of GMRES iterations allowed in each Newton iteration. Our computational experiments show that it is usually sufficient to limit the maximum number of GMRES iterations to 5. Furthermore, η_*k*_ can also be chosen in a dynamic way with η_*k*_ being relatively large for small *k* and relatively small for large *k*.

## 4. Comparison With DIIS

The DIIS method (Pulay, 1980) is a commonly used technique to accelerate the convergence of iterative method for solving the CC equation. At the *k*th iteration, we form a new approximation as

(28) $$\begin{array}{l} {\tilde{t}}^{\left(k+1\right)}=\overset{k}{\underset{j=k-\ell }{\sum }}\omega_{k-j}\left[t^{\left(j\right)}+\Delta^{\left(j\right)}\right], \end{array}$$

for some constant ℓ < *k*, where ω_*j*_'s are chosen to be the solution to the following constrained minimization problem

(29) $$\begin{array}{l} \underset{\sum_{j}\omega_{j}=1}{\min}\|\underset{j}{\sum }\omega_{j}\Delta^{\left(k-j\right)}\|. \end{array}$$

The *k* + 1st amplitude approximation is then computed from

(30) $$\begin{array}{l} t^{\left(k+1\right)}={\tilde{t}}^{\left(k+1\right)}-{\tilde{\Delta }}^{\left(k+1\right)}, \end{array}$$

where ${\tilde{\Delta }}^{\left(k+1\right)}$ is the approximate solution to the Newton correction Equation (20) or (25).

Note that in some formulations of the DIIS algorithm, the Δ^(*k*−*j*)^ term in the objective of Equation (29) are simply replaced by *r*(*t*^(*k*−*j*)^). Since ${\tilde{\Delta }}^{\left(k-j\right)}$ is often computed as *D*^−1^*r*(*t*^(*k*−*j*)^), where *D* is the diagonal component in Equation (26), these two formulations are equivalent up to a scaling matrix *D*.

In some implementations of the DIIS acceleration method, one performs a fixed number of IN iteration with *D* in Equation (26) as the approximate Jacobian matrix before the DIIS procedure is used to update *t*^(*k*+1)^ according to Equation (30). In other implementations, DIIS is performed in each IN iteration.

The convergence of the DIIS method and its connection with the Broyden's method (Dennis and Schabel, 1996) has been analyzed in Rohwedder and Schneider (2011) and Walker and Ni (2011). The connection between DIIS and Krylov subspace method is made in Harrison (2004) and Ettenhuber and Jrgensen (2015).

One of the practical issues one needs to consider when implementing the DIIS method is the solution of the constrained minimization problem (29). A commonly used approach in existing quantum chemistry software is to write down the linear equation representing the first-order necessary condition of Equation (29) and solve the equation using a Cholesky or LU factorization based method. This approach is not numerically stable, especially when the set of {Δ^(*k*−*j*)^} becomes nearly linearly dependent. A more stable way to solve Equation (29) is to turn it into a unconstrained least squares problem and obtain the optimal solution via a rank-reveal QR factorization of the matrix consisting of Δ^(*k*−*j*)^ as its columns. However, applying rank-reveal QR to {Δ^(*k*−*j*)^} is rather costly. In comparison, performing a rank-reveal QR factorization for solving the projected least squares problem (24) in the NK method is relatively straightforward, and does not introduce significant overhead. To improve numerical stability and computational efficiency, it may be necessary to keep only a subset of {Δ^(*k*−*j*)^}'s for a small number of *j*'s.

In terms of memory usage, NK is slightly more efficient. In addition to storing the current approximation to the CC amplitudes and the residual, NK also stores orthonormal basis tensors of the Krylov subspace used to obtain approximate solution to the Newton's correction equation. The DIIS method typically needs to store a set of {Δ^(*k*−*j*)^}'s as well as the corresponding set of previous amplitude approximations.

Although one can view the DIIS method as a way to solve the Newton correction Equation (20) (Rohwedder and Schneider, 2011), it is also possible to combine DIIS acceleration with the NK procedure. In such a hybrid scheme, we simply use GMRES to compute ${\tilde{\Delta }}^{\left(k+1\right)}$ correction in Equation (30) after ${\tilde{t}}^{\left(k+1\right)}$ is obtained from a DIIS update.

## 5. Results and Discussion

In this section, we present a few numerical examples to demonstrate the effectiveness of the NK method and compare with the inexact Newton method accelerated by DIIS. All algorithms compared below were implemented using the NWChem software (Valiev et al., 2010) version 6.6.

### 5.1. H_2_O Molecule

In the first example, we show how the NK algorithm behaves when it is applied to a simple water molecule in equilibrium. We use the cc-pvtz basis set to discretize the problem. We compare the NK algorithm with the IN method in which the Jacobian is approximated the diagonal matrix *D* in Equation (26), and the IN method accelerated by the DIIS procedure (labeled as DIIS). When DIIS is used to accelerate convergence, it is applied every 5 IN iterations. We set the convergence tolerance to 10^−7^, i.e., we terminate the IN, DIIS, and NK iterations when the Euclidean norm of the residual *r*(*t*) falls below 10^−7^.

**Figure 1:**
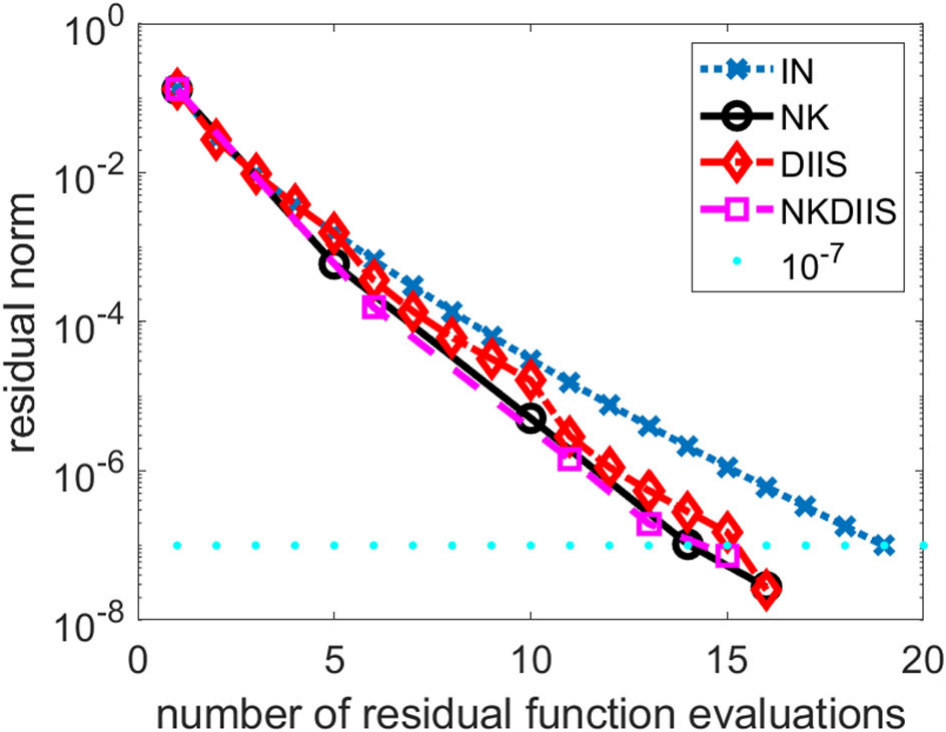
A comparison of different CCSD solvers for the H_2_O molecule.

In Figure 1, we plot the change of residual norm of each method with respect to the cumulative number of CCSD residual function evaluations (tensor contractions). Note that in the IN and DIIS runs, the number of function evaluations is equivalent to the number of IN iterations. However, in the NK run, the number of function evaluations is the total number of inner GMRES iterations and the number of outer NK iterations.

We observe that for this relatively easy problem, the IN method converges without DIIS acceleration. It takes 19 iterations (and 19 function evaluations) to reach convergence. The use of DIIS acceleration reduces the total number function evaluations to 16. This is also the number of function evaluations used in the NK method. By combining NK and DIIS, we reduce the number of functions by 1.

### 5.2. Cr_2_

In this section, we show how NK performs on a Cr_2_ molecular. The interatomic distance between two Cr atoms is set to 1.7 angstrom, which is near equilibrium. We use the cc-pvdz basis set to discretized the problem.

**Figure 2:**
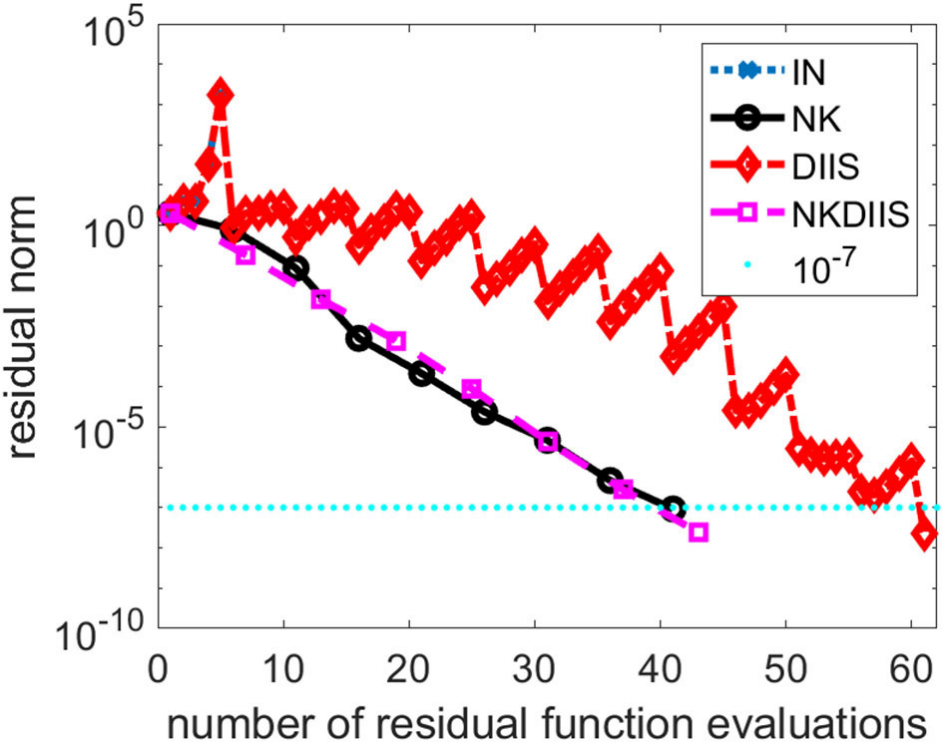
A comparison of different CCSD solvers for the Cr_2_ molecule.

This is a relatively difficult problem. As we can see from Figure 2, without DIIS acceleration, the IN iteration diverges quickly. Even when the DIIS acceleration is activated, which is applied every 5 IN iterations, the change of residual norm has a zig-zag pattern with the residual norm decreasing only after a DIIS step. It takes a total of 61 residual evaluations before convergence is reached. Both NK and the hybrid NK and DIIS converge rapidly. We used a maximum of 5 GMRES iterations in each NK iteration. The residual norm decreases monotonically in both runs. There is very little difference between the two.

**Figure 3:**
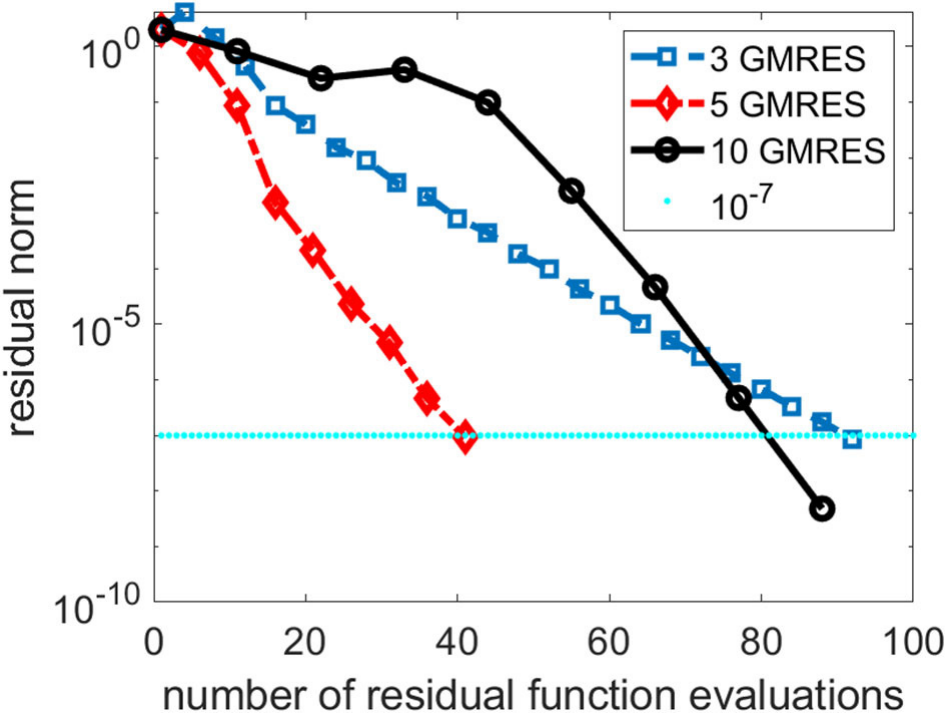
A comparison of three Newton–Krylov (NK) runs that use 3, 5 and 10 Generalized Minimum Residual (GMRES) iterations per NK iteration.

As we indicated earlier, there is a tradeoff between performing more GMRES iterations in the inner loop of the NK method and the number of NK iterations. In Figure 3, we compare the total number of NK iterations and the total number of function evaluations for several NK runs in which different numbers of GMRES iterations were performed in each outer NK iteration.

We can see from Figure 3 that performing 3 GMRES iterations per NK iteration is not enough to achieve rapid convergence. On the other hand, taking too many GMRES iterations does not help either, especially in the first few NK iterations when the residual norm is still relatively large. For this problem, setting the maximum number of GMRES iterations per NK iteration to 5 appears to yield best performance.

**Figure 4:**
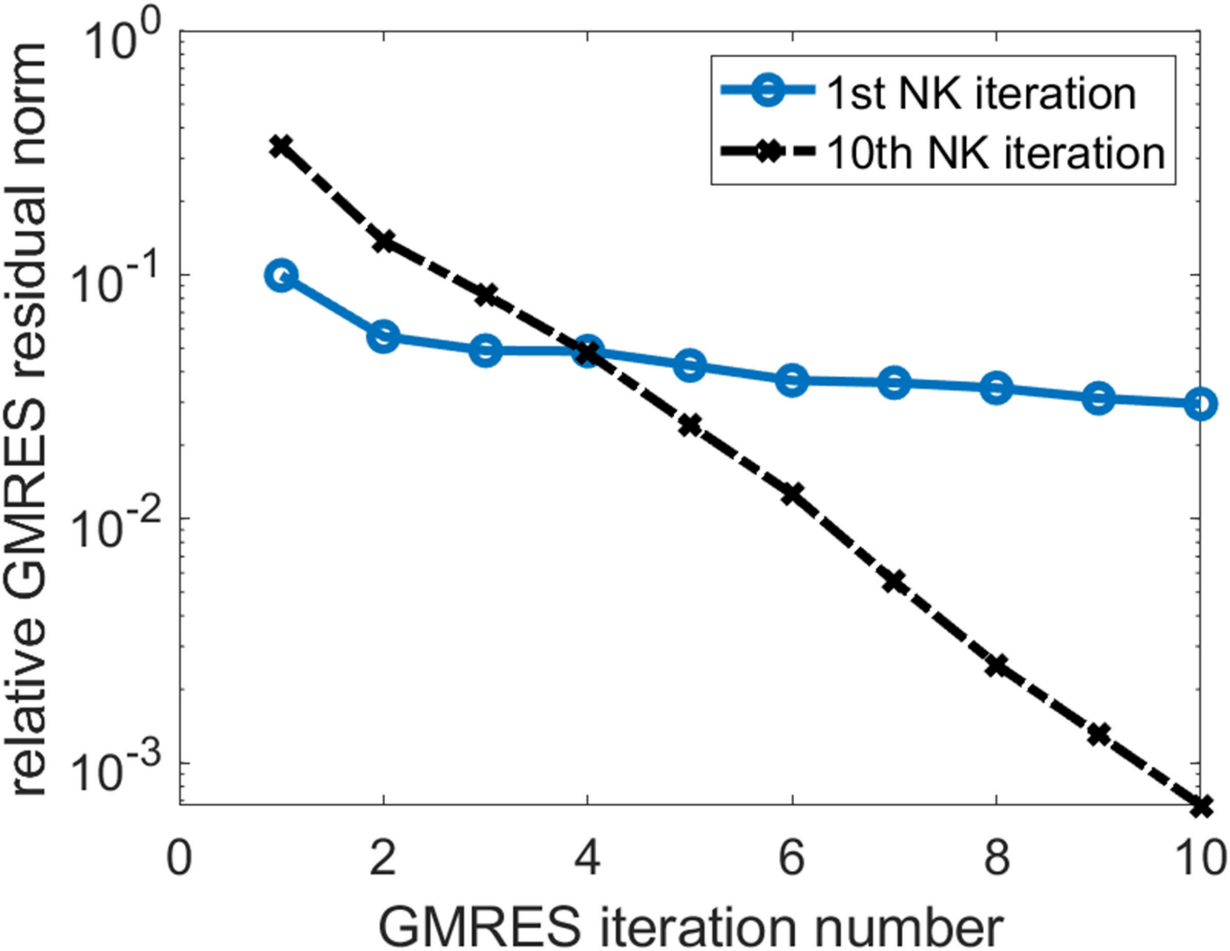
The change of relative GMRES residual norm in the first and 10th NK iterations.

In Figure 4, we also show the change of the relative GMRES residual norm defined as ||*Hs* − β*e*_1_||/β, where *H*, *s*, *e*_1_, and β are as defined in Equation (24), with respect to the GMRES iteration number during the first and the 10th NK iterations when the number of GMRES iterations is fixed at 10. We observe that the GMRES iteration converge slowly in the first NK iteration when the CCSD amplitudes are relatively far from the solution. As the amplitudes become closer to the solution, the GMRES iteration converges faster.

### 5.3. Trans-dimer Ti_2_O_4_

**Figure 5:**
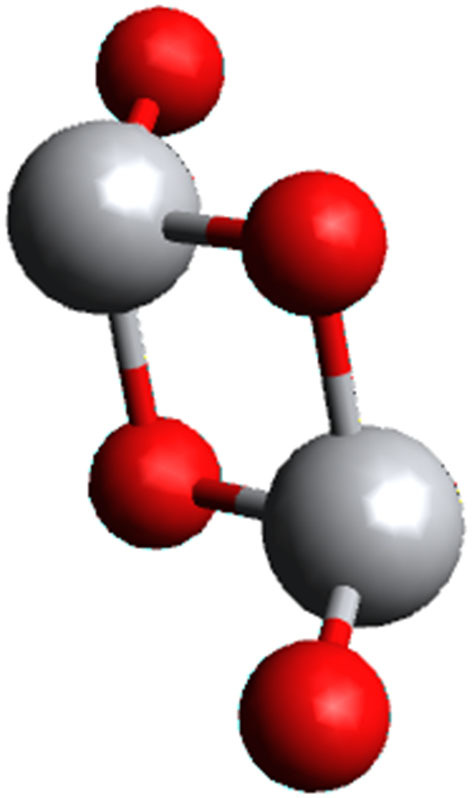
The atomic configuration of the trans-dimer Ti_2_O_4_ system.

The next example we use for testing the NK method is a titanium oxide system. Its geometry is shown in Figure 5. We use the aug-cc-pvtz basis set to discretize the problem.

**Figure 6:**
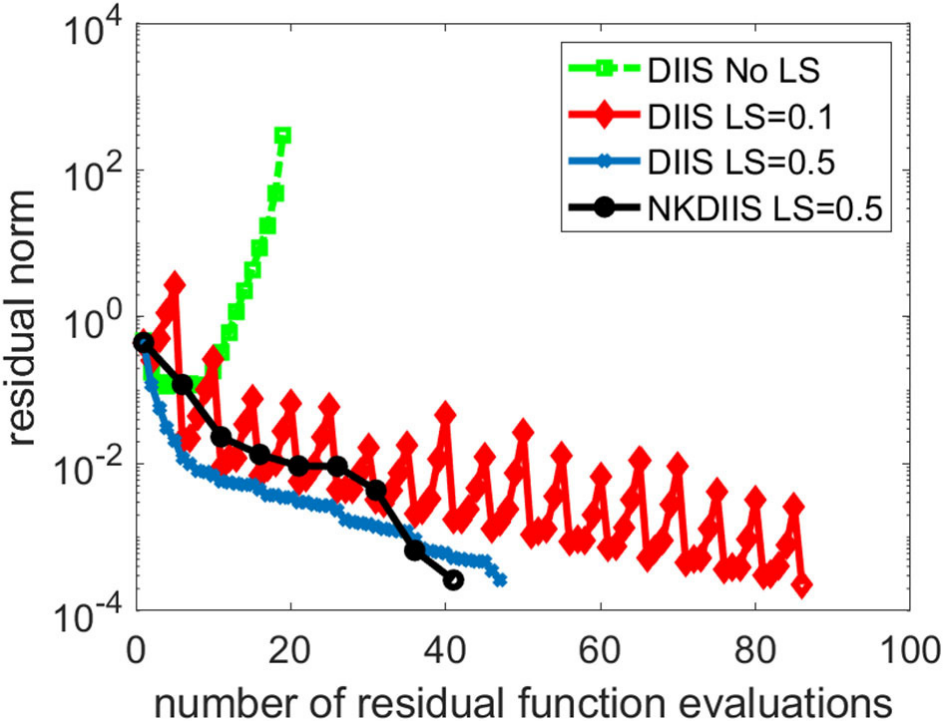
A comparison of different CCSD solvers for the trans-dimer Ti_2_O_4_ molecule.

This is a difficult problem. Figure 6 shows that DIIS fails to converge. In fact, the residual norm quickly increases. This is mainly caused by the fact that the *D* matrix used in the IN method is extremely ill-conditioned. To overcome this difficulty, we regularize the NK calculation by subtracting a constant shift σ from the diagonal of *D*. The same level-shifting is used in the IN accelerated by DIIS. Figure 6 shows that DIIS converges when σ is chosen to be 0.1. However, the convergence is rather slow with this choice of level shift. By setting σ to 0.5, we can achieve much faster convergence. The shift can also be applied to in NK when *D* − σ*I* is used as a preconditioner in the GMRES iteration. For this problem, the NK (combined with DIIS) converges with 41 residual function evaluations, which is fewer than the 47 function evaluations required in DIIS. Similar convergence is observed for NK without DIIS (which we do not plot here) also.

### 5.4. oxo-Mn (salen)

**Figure 7:**
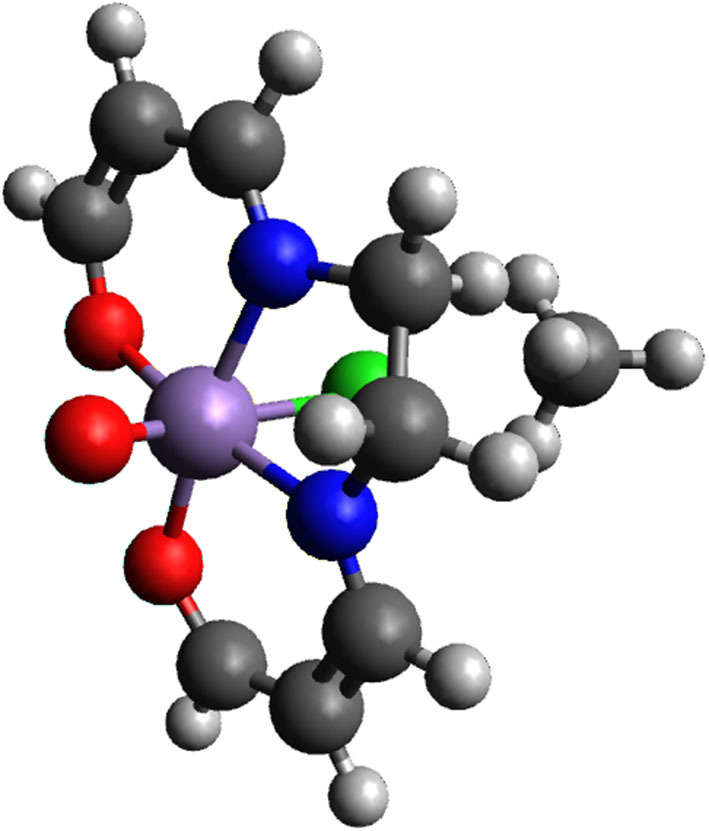
The atomic configuration of the oxo-Mn (salen) molecule.

We now compare the performance of NK and DIIS for an oxo-Mn (salen) molecule shown in Figure 7. In practical applications, this system catalyzes enantioselective epoxidation of unfunctional olefines and represents an important substance in industry. Due to the quasi-degeneracy of the lowest states, this system is a considerable challenge even for multireference methods and has been intensively studied recently (Irie et al., 1990; Zhang et al., 1990; Antalík et al., 2019).

We have performed TCCSD calculations in CAS(28,22) space, where external amplitudes were obtained from DMRG calculation.

**Figure 8:**
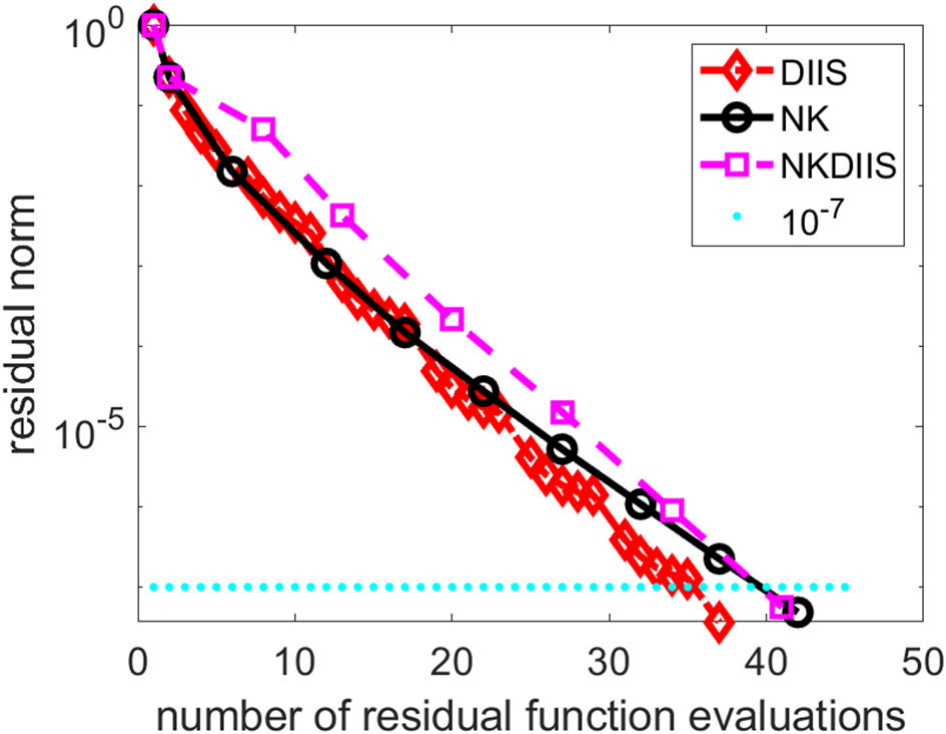
A comparison of direct inversion of iterative subspace (DIIS) and NK combined with DIIS (NKDIIS) convergence for the oxo-Mn (salen) molecule.

Figure 8 shows the convergence history of the IN iteration accelerated by DIIS, NK, and NK combined with DIIS (NKDIIS). For this example, the IN iteration appears to converge very fast. Both the NK and NKDIIS are slightly slower, but converges in 10 NK iterations. This example shows that for systems with multireference features, it is important to use an appropriate model that can treat the multireference character of the molecule effectively. With such a model, the CCSD nonlinear equation can become easier to solve. Although NK may not offer too much advantage in this case, it is still an effective solver for such as model.

### 5.5. The G2/97 Dataset

In addition to testing the NK algorithm on the above representative molecules, we performed a more extensive testing of the algorithm on a much wider range of molecules randomly selected from the G2/97 dataset (Curtiss et al., 1997). Among 130 systems we tested, NK performs better on 123 of them. On average, NK uses 12% fewer function evaluations when compared with DIIS. In the best case, NK uses 33% fewer function evaluations. In the worst case, NK uses 40% more function evaluations.

## 6. Conclusion

We presented a NK method for solving CC amplitude equations. In such a method, the Newton correction equation is solved by a Krylov subspace iterative method such as the GMRES method. Preconditioners can be applied in the iterative solver to accelerate convergence. We discussed the trade-off between performing more inner (GMRES) iteration and outer Newton iteration, and suggested an adaptive stopping criterion for the inner iteration. We compared the NK method with the widely used DIIS method and showed how the two methods can be combined. We presented several numerical examples to demonstrate the effectiveness and robustness of the NK method not only for standard CCSD calculations but also for tailed CCSD calculations where the information for external correction is obtained from a DMRG calculation. Although the results we presented in this paper are on developments made in an older version NWChem software, the NK has been implemented in the next generation of NWChem software (NWChemEx) (Richard et al., 2019) designed for exa-scale high-performance computing platforms.

## Data Availability Statement

The original contributions presented in the study are included in the article/supplementary material, further inquiries can be directed to the corresponding author/s.

## Author Contributions

All authors listed have made a substantial, direct and intellectual contribution to the work, and approved it for publication.

## Conflict of Interest

The authors declare that the research was conducted in the absence of any commercial or financial relationships that could be construed as a potential conflict of interest.
